# Using Generative AI to Co-Design Digital Mental Health Interventions With Adolescents in Rural South Africa: Qualitative Thematic Analysis of Participatory Workshops

**DOI:** 10.2196/73535

**Published:** 2025-12-05

**Authors:** Sophie Dallison, Bianca Moffett, Princess Makhubela, Tamera Nkuna, Julia R Pozuelo, Alastair van Heerden, Heather O'Mahen

**Affiliations:** 1 Department of Psychology, University of Bath, 10 West, Claverton Down, Bath, Somerset, BA2 7AY, United Kingdom, 4401392661000; 2 MRC/Wits Rural Public Health and Health Transitions Research Unit (Agincourt), School of Public Health, Faculty of Health Sciences, University of the Witwatersrand, Johannesburg, South Africa; 3 Department of Global Health and Social Medicine, Harvard Medical School, Harvard University, Boston, MA, United States; 4 Department of Psychiatry, University of Oxford, Oxford, United Kingdom; 5 Faculty of Health Sciences, University of the Witwatersrand, Johannesburg, South Africa; 6 Mood Disorders Centre, Department of Psychology, University of Exeter, Exeter, United Kingdom

**Keywords:** digital mental health, generative AI, co-design, adolescents, South Africa, participatory research, qualitative study

## Abstract

**Background:**

Digital mental health interventions (DMHIs) offer a scalable approach to address adolescent depression and anxiety. User-centered coproduction can optimize acceptability and engagement, but it is often resource-intensive. Advances in generative artificial intelligence (GenAI) create new opportunities for involving adolescents in co-design, yet research on its feasibility and acceptability, particularly in low-resource settings, remains underexplored.

**Objective:**

This study aimed to explore adolescents’ experiences and perspectives of using GenAI to co-design stories, images, and music for the Kuamsha app (Sea Monster), a gamified DMHI that teaches behavioral activation through interactive narratives and peer support.

**Methods:**

Overall, 2 participatory workshops and focus group discussions were conducted with 23 adolescents (aged 15‐19 years) in rural Mpumalanga, South Africa. Participants were guided to use 3 GenAI tools—ChatGPT (OpenAI), text-to-story; MidJourney (MidJourney Inc), text-to-image; and Soundful (Soundful Inc), music generation—to create digital content. Data were audio-recorded, translated, transcribed, and triangulated with the facilitator’s observation notes. Thematic analysis was used to explore key themes.

**Results:**

Almost all participants (22/23, 96%) had no prior exposure to GenAI. The majority (20/23, 87%) described the creative process as enjoyable and engaging, with most (21/23, 91%) reporting that creating music improved their mood. Adolescents expressed autonomy and ownership of the process, with more than half (14/23, 61%) personalizing outputs to reflect their identities and aspirations. All participants (23/23, 100%) preferred artificial intelligence (AI)–generated images over the cartoon-like illustrations of the Kuamsha app, and most (19/23, 83%) preferred AI-generated music. Story preferences were more mixed, with about a quarter of participants (6/23, 26%) recalling that Kuamsha’s narratives contained embedded lessons that were not integrated into the ChatGPT outputs. Most adolescents (18/23, 78%) required support with prompt construction, and more than half (13/23, 57%) noted cultural biases in AI outputs, particularly in images. Most participants (17/23, 74%) expressed interest in using AI for schoolwork and creative projects, while a minority (6/23, 26%) preferred to limit use to personal applications. Concerns about fairness and the displacement of human creativity were also raised.

**Conclusions:**

GenAI shows promise for enhancing adolescent engagement in the coproduction of DMHIs and enabling culturally relevant and personalized content. However, reliance on human support and persistent algorithmic biases remain limitations. Further research should explore the integration of therapeutic principles into AI-generated media and strategies to mitigate bias.

## Introduction

More than 90% of the world’s adolescents live in low- and middle-income countries (LMICs) where large treatment gaps for depression and anxiety prevail [1]. In rural South Africa, mental health services are scarce, leaving young people with limited access to care [2]. Digital mental health interventions (DMHIs) have emerged as a promising opportunity to bridge this gap, offering anonymity, flexibility, and the potential to reach large numbers of adolescents at low cost [3,4]. Despite their promise, engagement remains a persistent challenge and an important determinant of intervention effectiveness [5]. Key factors associated with higher engagement include the relatability of situations and characters, appealing aesthetic features, and age-appropriate design [6-8]. However, much of the existing DMHI content has been developed in high-income countries [6-9], limiting its cultural resonance elsewhere.

To improve cultural fit and engagement, co-design approaches, whereby potential users play an active role in the intervention’s development, have been increasingly adopted [10,11]. Such participatory methods help ensure that interventions reflect users’ experiences and preferences, positioning adolescents as “experts of their own experiences” [8]. However, co-design is resource-intensive, often requiring multiple iterative workshops with creative teams to generate narratives, images, and other materials [10,11]. This limits the scalability of DMHI development, particularly in low-resource settings where capacity and funding are constrained.

Artificial intelligence (AI) is now being integrated into DMHIs in diverse ways, including predicting risk for mental health conditions, informing treatment selection, monitoring progress through wearable data, creating chatbots that deliver therapy, and improving care quality [12,13]. To date, however, most applications have focused on clinical support or content delivery rather than user cocreation. Recent advances in generative artificial intelligence (GenAI), including large language and diffusion models capable of creating text, images, and music from simple prompts, offer a new avenue for participatory design [12,13]. By embedding GenAI within participatory design frameworks [14,15], developers could make the co-design of DMHIs faster, more creative, and more scalable while maintaining the core principle of user-driven innovation. At the same time, integrating AI into global mental health design raises important ethical and cultural considerations. Most GenAI models are trained on data that overrepresent Western, educated, industrialized, rich, and democratic contexts, leading to outputs that may be biased or culturally incongruent [16]. Engaging adolescents from LMICs in participatory exploration of these tools offers a way to identify such biases early and ensure that future digital interventions better represent diverse cultural perspectives.

This study builds on the Digital Delivery of Behavioural Activation Therapy to Overcome Depression and Facilitate Social and Economic Transitions of Adolescents in South Africa (DoBAt) pilot trial, which evaluated the feasibility, acceptability, and initial efficacy of digitally delivered behavioral activation (BA) therapy among adolescents with depression in rural South Africa [17]. Adolescents in the intervention arm received BA therapy via the Kuamsha program, comprising a smartphone app (the Kuamsha app) and peer mentor support calls. The Kuamsha app was a coproduced, interactive choose-your-own-adventure narrative game that delivered the main principles of BA through a gamified story format. The app comprised 2 interactive user-led stories, one about a school song contest and one about a football match, each divided into 6 sequential episodes, engaging users through thoughtfully crafted content, images, and music. The Kuamsha app and its components, including stories, images, and music, were developed through an iterative process of co-design involving a series of participatory workshops, focus group discussions, and individual interviews [18] to ensure the content was appealing and relatable. However, the design process was lengthy and resource-intensive, particularly writing and visualizing the stories. The 2 stories, while engaging, had limitations in scope. Feedback from adolescents further revealed that they had a desire for more content diversity and depth.

Given these constraints and AI’s growing potential to improve participatory design, this study examined whether GenAI could make co-design faster and more scalable without sacrificing quality or cultural fit. Through participatory workshops and focus groups with rural South African adolescents, we explored their experience using GenAI tools to create stories, images, and songs, comparing these outputs with the original Kuamsha content. This approach allowed us to assess whether AI could meaningfully support future DMHI development while preserving the user-centered, culturally grounded approach that makes interventions engaging and effective.

## Methods

### Setting and Recruitment

This study was conducted in the Bushbuckridge subdistrict of Mpumalanga Province, South Africa, a predominantly low-income area with high levels of youth unemployment and limited access to specialist mental health services. The South Africa Medical Research Council/University of the Witwatersrand (MRC/Wits) Rural Public Health and Health Transitions Research Unit (Agincourt) maintains a long-standing health and sociodemographic surveillance system supporting community-based and clinical research in the area. Workshops were held at the South Africa MRC/Wits-Agincourt facility within the study site.

Participants were drawn from the intervention arm of the DoBAt pilot trial, which evaluated the feasibility, acceptability, and preliminary efficacy of the Kuamsha app, a mental health app that delivers BA through a narrative game supported by peer mentors. Adolescents eligible for this substudy were those who had previously received Kuamsha as part of the DoBAt trial and who reported being comfortable reading, writing, and speaking English (as the primary local language is Shangaan and Xitsonga, and at the time of data collection, no GenAI tools were available that could operate effectively in this language).

Recruitment was carried out by an experienced fieldworker (PM) from the MRC/Wits-Agincourt team, who had previously supported data collection for DoBAt. Potentially eligible participants were visited at their homes, where the study objectives, procedures, and ethical considerations were explained in both English and Xitsonga. Interested adolescents and caregivers of those younger than 18 years were provided with information sheets and asked to provide written informed consent. Recruitment continued until 23 participants aged 15‐19 years were enrolled, representing a purposive, nonprobabilistic sample.

### Materials

Overall, 3 GenAI tools were used in this study. ChatGPT (gpt-3.5-turbo-0301; OpenAI) [14], a language model for generating stories, MidJourney (version 4.0), a generative text-image AI developed by MidJourney, Inc [19] for generating images, and Soundful (Soundful Inc), an AI music generative platform [20] for composing songs. ChatGPT and MidJourney operate on text-to-text and text-to-image bases, requiring users to input prompts, while Soundful allowed participants to create music by selecting options from its user interface (the text-to-music functionality was not available in a user-friendly format at the time of conducting this study, although such features have since been developed).

### Procedure

#### Overview

All participants, along with caregivers of those younger than 18 years, provided written informed consent, including consent to audio-record the workshops. The research team prepared resources for the workshop, including a presentation. Qualitative focus group discussion topic guides were developed by SD, JRP, and AvH. Prior to the workshops, the research assistant (SD) provided training to 3 qualitative fieldworkers (TN, PM, and Meriam Meritze) on using the 3 AI tools and to pilot the topic guides.

#### Participatory Workshops

In total, 2 day-long workshops were conducted on consecutive Saturdays in April 2023 to avoid interfering with school hours. The workshops were facilitated by SD as well as TN, PM, and Meriam Meritze, who are fluent in English and Xitsonga (the local language).

The workshop began with SD giving a presentation to introduce participants to GenAI, explain its potential uses, and demonstrate the 3 tools they would be using in the workshop (ChatGPT, Midjourney, and Soundful).

Participants were then divided into 3 groups of 3‐4 individuals, each facilitated by 1 fieldworker who supported and encouraged the generation of ideas. Each group had access to 1 laptop to use the AI tools, with all groups working on the same AI tool simultaneously. SD provided additional support by answering questions, encouraging participation, and assisting with technical issues. An IT technician was also present to resolve any data, network, or more complex technical issues.

The workshop activities were structured as follows. First, participants used ChatGPT to create stories. They were asked to include a character name, location, and activity in their prompts (eg, “Please write a story about a young South African boy playing football in the village”). Participants were also shown how to modify elements of the AI-generated stories by giving prompts, such as “Can you make the story more action-packed and engaging?” or “Can you change the name of the main character to a South African name?” or “Can you change X part of the story to Y?” Second, participants used MidJourney to create images. Participants were asked to be as detailed as they could with the prompts (eg, “A young African boy wearing a baseball cap in the style of a Marvel comic”). Examples of AI-generated images are mentioned in Multimedia Appendix 1. Third, participants used Soundful to create music on their website user interface. Participants were asked to choose the music genre, subgenre, key, speed, major or minor chord, and name their song.

#### Focus Group Discussion

After all groups had generated their own stories, images, and music using the AI tools, participants reconvened for the second part of the workshop: the plenary focus group. The focus groups were facilitated by TN with assistance from SD, PM, and Meriam Meritze. The purpose of the focus group was 2-fold: (1) to explore the adolescents’ experience using the 3 AI tools and (2) to compare the AI-generated stories, drawings, and songs with those created by humans for the Kuamsha app. Comparison content from the Kuamsha app, as well as the AI-generated media produced during the workshop, was shown on a projector screen to ensure that all participants could clearly see and hear the material.

A combination of English and Xitsonga was used throughout the workshops in order to facilitate the understanding and engagement of all participants. SD delivered the presentation in English, with PM providing translation into Xitsonga. Xitsonga was predominantly used during the small group breakaway sessions, and the focus group discussion was conducted in Xitsonga (although some participants responded in English). The workshops and the focus groups were audio-recorded.

This study was designed and reported in accordance with the Consolidated Criteria for Reporting Qualitative Research (COREQ) checklist for interviews and focus group discussions (Checklist 1). We also drew on the Guidance for Reporting Involvement of Patients and the Public to reflect the participatory co-design elements of adolescent involvement.

### Analysis

The audio recordings of the workshops and focus group discussions were translated from Xitsonga to English and transcribed by TN and PM. The transcripts were uploaded to NVivo (Lumivero) [21], and data were analyzed using inductive thematic analysis. SD reviewed the transcripts several times to familiarize themself with the data before creating the initial codes. SD discussed these initial codes iteratively with HO’M, AvH, and BM and then created final themes after team consensus. BM met with TN, PM, and Meriam Meritze to review the themes. Given the exploratory nature and small sample, we did not aim for thematic data saturation but rather sought to capture a breadth of adolescent perspectives.

### Reflexivity

The research team consisted of 7 researchers, who identified as female, and 1 researcher who identified as male, from a wide range of career stages. SD is a research assistant, TN, PM, and Meriam Meritze are experienced qualitative fieldworkers, BM (PhD and MD) is a clinician researcher, JRP (PhD) is a global mental health researcher, AvH (PhD) is a research psychologist and study co-principal investigator, and HO’M (PhD) is a professor of clinical psychology and study co-principal investigator. All members of the research team have prior experience conducting qualitative research. SD identifies as White British; TN, PM, and Meriam Meritze identify as Black African; AvH and BM identify as White South African; JRP identifies as White European; and HO’M identifies as White American. Members of the research team (SD, PM, Meriam Meritze, and TN) who facilitated the participatory workshops and conducted analysis were mindful of their positioning, cultural background, and prior exposure to digital technology and GenAI. We strived to create a fun, engaging, and inclusive environment to facilitate participants’ creative process. However, the participatory nature of the workshops, where facilitators were actively involved with the participants, meant that there was potential for power dynamics and personal biases to affect data collection.

### Ethical Considerations

This study was reviewed and approved by the Human Research Ethics Committee (Medical) of the University of the Witwatersrand, Johannesburg, South Africa (MED20-05-011). All participants provided written informed consent before enrollment. For participants younger than 18 years, written parental consent was additionally obtained. The informed consent process included information on the study objectives, procedures, confidentiality, and the voluntary nature of participation, including the right to withdraw at any time without consequence. Participants did not receive monetary compensation. Instead, they were provided with transport to the study venue, refreshments (tea and lunch), and reimbursement for travel-related expenses. Workshops were audio-recorded, transcribed, and translated into English. Identifying details were removed during transcription, and transcripts were stored securely on password-protected servers. All reported data are anonymous and deidentified. Names have been changed to protect participants’ privacy. Furthermore, no images in the article or supplementary material contain identifiable information about individual participants. Care was taken to ensure that any visual material generated during the workshops (eg, AI-produced images) did not depict or identify real individuals.

## Results

### Overview

Adolescents described themes that encapsulated their experience of using GenAI tools during the workshop, including the “process of generating media,” “emotional experience,” and “autonomy and agency” of creation. They also reflected on the media they had created under the theme “resonance with AI-generated media” and compared their creations to the Kuamsha app under *“*Kuamsha versus AI comparison.” Finally, adolescents considered the broader implications of AI under the themes “morality of AI” and “adolescents’ use of AI in the future.”

Quotes marked with an asterisk (*) indicate that they are from the workshops when adolescents were using the AI tools, and those without are from the focus group discussions.

### Support With Prompt Engineering

Given that most adolescents (22/23, 96%) had no prior exposure to GenAI, facilitators provided simple guidance on how to structure prompts (ie, “prompt engineering”). Adolescents were encouraged to begin with a subject, an action, and a setting (eg, “a young South African boy playing football in the village”). Fieldworkers supported participants in refining prompts to make them more culturally relevant or more engaging.

Examples of prompts used with the GenAI tools included:

Write a story about a young South African boy named Admire playing soccer in Bushbuckridge.

[Prompt for ChatGPT]

A young Black South African girl in school uniform, in the style of a Marvel comic.

[Prompt for MidJourney]

Afrobeat, 4/4 rhythm, upbeat.

[Prompt for Soundful]

Adolescents’ proficiency in English also varied, and some adolescents required support from peers or facilitators to translate their prompts into English.

We have ideas, but we don’t know how to put it.

[Participant #16]

Tell me in Xitsonga and I will translate to English for you.

[Fieldworker #3]

Like, I just don’t know how to construct my sentence.

[Participant #16]*

### Usability

Once participants had started using the tools, they described the process as easy and enjoyable, and they understood the tools by actively using them. Furthermore, when facilitators asked about participants’ understanding of ChatGPT, they were able to provide an accurate description. Participants were also quick to recognize ChatGPT’s ability to:

Pick up that you forgot to type something in.

[Participant #6]

It was difficult in the beginning but the more we did it the more it became easy.

[Participant #9]

It was very easy.

[Participant #20]

It was fun.

[Participant #17]

### Mood and Emotional Experience

The majority (20/23, 87%) described the process as enjoyable and engaging, with most (21/23, 91%) reporting that creating music improved their mood.

It’s our first time doing this and we are loving it.

[Participant #4]*

You’re even dancing to the beats.

[Facilitator]*

When I play the music I made myself, it can make me feel good about myself, it can improve my mood or relieve the stress.

[Participant #22]

### Autonomy and Agency of Creation

The freedom adolescents had to create was a prominent theme. Adolescents expressed autonomy and ownership of their creations.

I am the producer; everyone likes their own creation.

[Participant #21]

We get the freedom to do what we like on the music that we make ourselves.

[Participant #1]

### Personalization of Outputs

Adolescents’ prompts often reflected their own characteristics, interests, hopes, and aspirations and were supported to refine prompts to make them more culturally relevant or engaging.

I chose that topic because I love playing soccer and I wanted to understand what it takes or to be a good player and what needs to be done to qualify for the tournaments.

[Participant #3]

I want to be an IT specialist in future and my topic was based at that and the story that came out inspired me because it started from when the character on the story was still dreaming of becoming an IT specialist right to the end where his dream came true.

[Participant #4]

I wanted it to generate Miss Universe pictures because I aspire to be a model one day when I finish school; so I wanted to see if I would be able to get their pictures using that app.

[Participant #1]

### Using Prompt Engineering to Change Elements of the Story

Most adolescents (18/23, 78%) required support with prompt construction; however, as the workshop progressed, adolescents and facilitators began to experiment with prompt engineering to adapt features of the story.

Let’s prompt it to change the name of the character to Admire.

[Participant #7]*

We started from bullying and we moved to supportive, let’s try to make it more exciting and more positive.

[Fieldworker #3]*

### Resonance With AI-Generated Media

The overwhelming feeling from participants was how realistic the AI-generated images were, with regard to how life-like or photographic the images looked. However, more than half (13/23, 57%) noted cultural biases in AI outputs, particularly in images.

These images look real and I think I have seen one on TV before.

[Participant #6]*

When you give it the prompts of what you are looking for; it was able to generate those images. For example, I was looking for images of football players and it generated exactly that.

[Participant #5]

I wanted it to generate pictures of houses in the villages but the ones I have seen were completely different from our village house… Maybe that is how village houses in other countries look like.

[Participant #8]

Facilitators supported adolescents to explicitly write prompts which included “African,” “South African,” or “Black,” for MidJourney to produce an image that would be culturally accurate to the prompt and work around Western, educated, industrialized, rich, and democratic (WEIRD) biases.

I didn’t want a White person.

[Participant #18]

You should have been specific about that. If you want an African, you should mention that.

[Fieldworker #4]*

### Kuamsha Versus AI-Generated Content

#### Stories

Compared to the Kuamsha stories, adolescents enjoyed having ownership over the stories created using ChatGPT, such as creating the characters and choosing the storyline. However, 1 adolescent reflected that they liked the lessons taught in the Kuamsha story, while this was not mentioned about the ChatGPT stories.

In the story I made myself; I was able to give it prompts of how I want the story to be like and it was writing the story exactly how I have prompted it.

[Participant #2]

I personally enjoyed the story from the app because there were different characters and throughout their journey in the story, there were lessons that they were teaching each other and as a reader I was also learning from those lessons.

[Participant #5]

Furthermore, some participants found the English used by ChatGPT too complex and above their level, while others found it easier to understand than the language used in the Kuamsha app.

The *Kuamsha* language was simple. There were some words that were difficult to understand on the story that we made [using AI], and I even thought that maybe we should have a dictionary in order to understand them.

[Participant #6]

Sometimes I had to read the *Kuamsha* story many times before I could understand some parts of the story, but with the one I made I can even easily explain it to someone else.

[Participant #23]

#### Images

All participants preferred the images they had generated using MidJourney to those used in the Kuamsha app as they were deemed more realistic and less cartoon-like.

I prefer the ones I made because they are realistic compared to the *Kuamsha* ones. The ones from *Kuamsha* are just cartoons.

[Participant #23]

#### Songs

Most participants preferred the music generated with AI to the Kuamsha music.

[I prefer] the one I made myself because I feel like the *Kuamsha* music is made for toddlers and the beats they used are not that for the adolescents like us; kids would enjoy that music.

[Participant #3]

### Morality of AI

When asked about the morality of GenAI to produce stories, images, and music, adolescents had varied responses. Most participants felt it was morally acceptable for GenAI to make a story, due to the perceived better accuracy of a computer over a human. However, 1 participant expressed concern over the ownership of the work and how they would not be learning from this process.

The AI is very good and it does exactly what you have asked it to do but personally I did not like it because I only prompt it with a topic and it does the whole job for me and I am not learning anything from it because I am not the one who did the job.

[Participant #6]

Regarding the morality of using GenAI for creating images, the majority of adolescents felt that it was morally unacceptable due to the potential disadvantage to artists.

I think it is a bad thing for artists because people can use the computer to generate the pictures that they want.

[Participant #17]

When asked about the morality of creating music using a user interface such as Soundful, adolescents felt that it was acceptable because they were simply selecting the genres and settings. However, when presented with a real-world example of AI being used to create music using existing artists’ voices, adolescents overwhelmingly felt this was morally unacceptable.

### Adolescents’ Use of GenAI in the Future

Most adolescents expressed an interest in using GenAI tools for both schoolwork and personal use.

Let’s say I have a school project to do, I can use [ChatGPT] and get good scores and it saves time because it does things quickly with less errors.

[Participant #17]

I would use [MidJourney] to search for pictures and I can’t print them out, I would just try to draw them myself by looking at the pictures generated using the MidJourney app.

[Participant #5]

A minority of adolescents’ views were strongly in the opposing direction, stating that they would not use AI tools for school but would consider using them for personal use.

It doesn’t help getting good grades for something that I did not do. Let’s say they ask me to tell them what I have written; I wouldn’t be able to tell them because I am not the one who wrote it.

[Participant #6]

I would use it to make stories that I can read for fun; I wouldn’t use it for my schoolwork.

[Participant #8]

## Discussion

### Principal Findings

This study reports a thematic analysis of an early-stage qualitative investigation to explore the feasibility and potential of using GenAI in the co-design and adaptation of a DMHI with adolescents in a low-resource context in rural South Africa. Participatory workshops were held to explore adolescents’ experiences using 3 GenAI tools (ChatGPT, MidJourney, and Soundful) to generate stories, images, and songs. Focus group discussions further explored adolescents’ experiences and compared the AI-generated outputs with those from the Kuamsha app, an interactive narrative game delivering BA created by humans without AI.

For all but 1 participant, the workshops were the first time they had encountered GenAI and been given the opportunity to explore how it works. Overall, adolescents found using GenAI to create stories and images with prompts and create music with an AI-based user interface to be an active, very engaging, and enjoyable process. These findings support the principles of BA, which emphasize the importance of supporting individuals with depression to undertake activities that are meaningful and rewarding. Adolescents liked aspects of the Kuamsha app that were self-relevant and, therefore, were particularly eager to generate stories, images, and songs with AI that were also self-relevant. For instance, adolescents chose prompts for stories and images mainly based on their interests and hobbies, aspirations for the future, naming the characters after themselves and peers, and creating music in their favorite genres. They commented on how this increased their connection with the created product; comments that are consistent with the Self-Determination Theory, which has shown that internal motivation increases when individuals are able to act in line with personally important interests and values [22].

A key challenge was that adolescents required support with prompt engineering. This was partly due to their unfamiliarity with AI, as this was a novel concept to participants at the time of the study. We also observed that support from facilitators was required to enable adolescents to exercise their creative abilities and fully participate in the project. We anticipate that some level of human support or facilitation would likely be required to ensure the quality and relevance of the content produced.

As hypothesized a priori, the creative process itself was a prominent theme to emerge from the workshops. Adolescents described feeling a sense of ownership over their outputs, being able to express themselves, and that the AI tools gave them freedom of creation. Despite the significant draw for adolescents, they did require support to exercise their creative abilities. This may reflect Albert and Runco’s position [23] on the balance of autonomy in creation: too much autonomy, and there is no focus, while the right amount can be good for creativity. Consistent with the literature on creativity and flow, an ideal amount of effort and challenge is required to achieve “flow state” [24]; thus, we can postulate that when adolescents had the correct balance between autonomy and support, they were able to engage in the creative process.

A key strength of this participatory study was that it captured the emotional and experiential benefits of generative creation. Adolescents described the process of creating stories, images, and especially music with AI as enjoyable, mood-enhancing, and empowering. Many reported feeling a sense of ownership and pride, with some even dancing to the music they had produced. Participants often became focused and absorbed in the task, qualities associated with improved learning [25]. Creating self-relevant media (stories, images, and songs) was intrinsically rewarding, helping adolescents to stay motivated and engaged in the task while also improving their mood. This suggests that involving adolescents in the development of the DMHI could lead to more engaging and effective treatments. Furthermore, the cocreation process is intrinsically reinforcing and mirrors the concepts BA is trying to teach (eg, engaging in valued activities in a focused and goal-directed manner). Beyond its value in coproducing culturally relevant content for DMHIs, our findings suggest that the act of generative creation itself may hold intrinsic therapeutic benefit, an innovative and underexplored avenue for digital mental health research in LMICs.

This study also revealed a significant Western bias in the GenAI models, particularly with the text-to-image model, MidJourney. Opportunities for reducing cultural biases in GenAI require both technical and participatory strategies. One approach is to diversify the training data on which large language and image models are built. Current models are predominantly based on data from Western, industrialized contexts, which often results in default outputs that fail to represent Global South populations. Expanding training datasets to include African languages, imagery, and cultural references would improve the ability of AI systems to generate outputs that better reflect local realities. Fine-tuning models on region-specific features, or developing smaller, locally trained models, could help align outputs with the sociocultural context of intended users. Human facilitation can also play an important role. For example, culturally attuned facilitators can assist users in crafting prompts that specify relevant cultural features (eg,“Xitsonga traditional wedding dress”). In addition, participatory data stewardship (where local communities contribute to and retain agency over the data used to train models) will be essential to ensure ethical inclusion. Finally, open access, local model development, and continuous bias monitoring are needed to ensure that GenAI evolves as a tool for inclusion and empowerment, enabling adolescents and other underrepresented groups to see their cultures authentically reflected in digital innovation.

However, when the AI tool did produce media that represented them, such as using character names similar to theirs or generated images with which they could identify, adolescents felt a strong sense of self-relevance and relatability, surpassing their connection to the more cartoon-like images in the Kuamsha app images. These findings are consistent with nascent literature demonstrating that young people tend to be more engaged with DMHIs when the characters are relatable and when the interface has appealing aesthetic features and is age-appropriate. The development of large language models (LLMs), including text-to-image, is happening at such an exponential pace that there are concerns it is outpacing the understanding of the biases within such systems [26]. Thus, there is a risk of perpetuating negative stereotypes and contributing to a process of othering and discrimination through AI, an issue that has been acknowledged by critics and developers of LLMs [26,27]. Urgent, yet thoughtful consideration needs to be taken by AI developers to correct this (eg, increasing use in the Global South and modifying algorithms), to reduce the potential for harm and to ensure that LLMs are not amplifying stereotypes. To avoid the perpetuation of existing cultural biases into GenAI tools, there is a pressing need to expand data on the use and implementation of GenAI, generally and within the context of DMHIs, in populations in LMICs.

### Limitations

This study was novel in the current global context of GenAI’s ubiquity and, to the authors’ knowledge at the time of conducting the study, was the first piece of research exploring adolescents’ use of and views on AI in a rural low- to middle-income context. However, there are some limitations that should be noted.

One limitation of this study is that the same adolescents who had previously used the Kuamsha app were also the participants who generated and evaluated AI content. This design was chosen intentionally to capture participants’ reflections based on first-hand creative experience, but it may have introduced bias into their comparisons. Participants could have been predisposed to prefer their own AI-generated creations due to a sense of personal ownership and self-expression, rather than because the content was inherently more appropriate. Conversely, their prior familiarity with Kuamsha, and whether or not they experienced improvement in their mood following its use, may have shaped their expectations and influenced how they judged AI outputs. As such, it remains unclear whether adolescents’ preference for AI-generated content reflected genuine superiority in cultural resonance or simply a stronger sense of personal investment. Future research should consider involving independent adolescent groups, or triangulating across multiple samples to reduce this bias and more robustly assess comparative preferences.

Second, this study was designed to explore adolescents’ experiential engagement with GenAI rather than to test its application within a therapeutic co-design process. As such, unlike the Kuamsha app, the stories, images, and music generated were not intentionally embedded with BA principles or other evidence-based psychological strategies. It is possible that a guided co-design approach, in which prompts explicitly incorporate therapeutic elements (eg, goal setting, problem-solving, or positive reinforcement), could generate content more directly aligned with intervention objectives. However, our primary objective was to assess whether GenAI could meaningfully engage adolescents, a necessary first step before adding therapeutic content. For this reason, we consider the absence of explicit BA elements a minor limitation, as it does not detract from the study’s aim to understand how adolescents interact emotionally and creatively with AI. Future work should examine how psychological support principles can be systematically integrated into AI-assisted co-design to maximize both engagement and clinical relevance.

Finally, while this study aimed to explore the use of GenAI, the tool used for generating songs was not text-to-audio due to the limited availability of such technology at the time. Future research will benefit from rapidly advancing multimodal AI models, which now offer sophisticated text-to-audio capabilities.

### Conclusions

Designing DMHIs has become more “humanized,” shifting from expert-led to participatory co-design with users [6]. This study explored whether GenAI could take this one step further, combining the contribution of humans and AI to improve engagement with DMHIs even more. Adolescents enjoyed generating stories, images, and songs and generally preferred the AI-generated content over that of the Kuamsha app. The creative process itself was an important part of the process, giving adolescents freedom of expression to create self-relevant media. However, adolescents require support to use the technologies and exercise their creative abilities. GenAI offers a transformative opportunity for developing DMHIs in resource-constrained settings. Unlike traditional co-design, which requires extensive workshops and specialized personnel, AI-assisted approaches enable adolescents to create culturally relevant content using natural-language prompts, producing multiple intervention scenarios in a fraction of the time and cost. This efficiency supports cross-cultural adaptation: the same tools can be deployed across diverse LMIC contexts, allowing local participants to generate region-specific materials rather than relying on interventions developed in high-income countries. Establishing shared repositories of culturally validated prompts and outputs could foster regional collaboration and accelerate the creation of locally grounded interventions. By democratizing and decentralizing the development process, GenAI provides a scalable pathway to culturally attuned mental health support, provided its use remains guided by ethical, inclusive, and context-sensitive principles.

## Supplementary material

10.2196/73535 Multimedia Appendix 1 Examples of artificial intelligence–generated images.

10.2196/73535 Checklist 1 COREQ checklist.

## Abbreviations

AI: artificial intelligence
BA: behavioral activation
DMHI: digital mental health intervention
DoBAt: Digital Delivery of Behavioural Activation Therapy to Overcome Depression and Facilitate Social and Economic Transitions of Adolescents in South Africa
GenAI: generative artificial intelligence
LLM: large language model
LMIC: low- and middle-income country
MRC/Wits: Medical Research Council/University of the Witwatersrand
