# Supplementary figures and images for: Using Generative AI to Co-Design Digital Mental Health Interventions With Adolescents in Rural South Africa: Qualitative Thematic Analysis of Participatory Workshops
Source: J Med Internet Res. 2025 Dec 5;27:e73535. doi: 10.2196/73535 (PMC12680127; doi:10.2196/73535)

**Figure S1.**


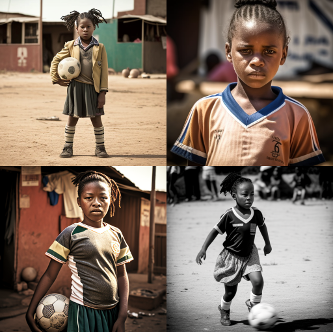


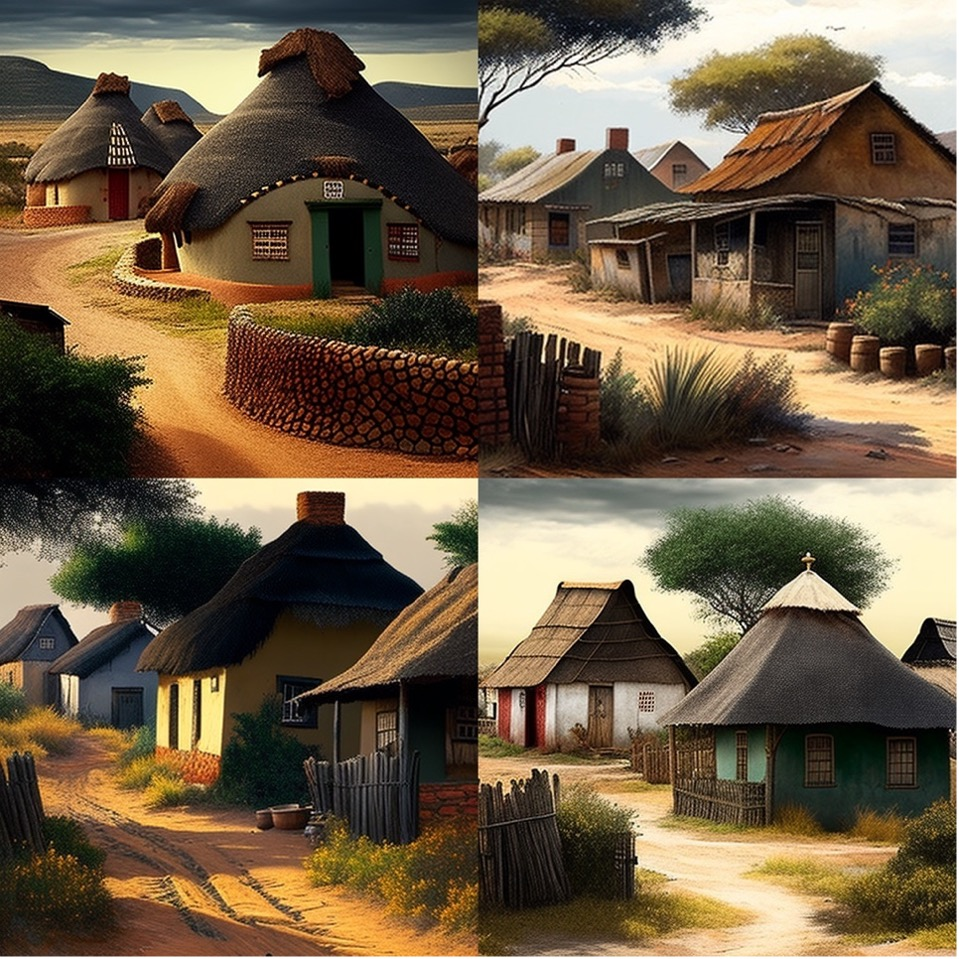


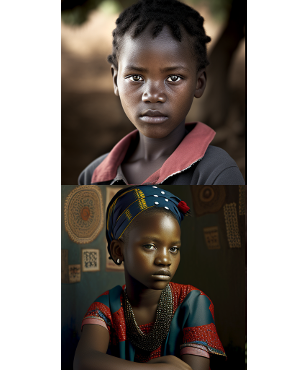

Supplement: Multimedia Appendix 1 [file jmir-v27-e73535-s001.docx]
